# Autophagic flux inhibition enhances cytotoxicity of the receptor tyrosine kinase inhibitor ponatinib

**DOI:** 10.1186/s13046-020-01692-x

**Published:** 2020-09-22

**Authors:** Diana Corallo, Fabio Pastorino, Marcella Pantile, Elena Mariotto, Federico Caicci, Giampietro Viola, Mirco Ponzoni, Gian Paolo Tonini, Sanja Aveic

**Affiliations:** 1 Neuroblastoma Laboratory, Fondazione Istituto di Ricerca Pediatrica Città della Speranza, Corso Stati Uniti 4, 35121 Padova, Italy; 2 Laboratory of Experimental Therapies in Oncology, IRCCS Istituto G Gaslini, Genoa, Italy; 3 Department of Woman’s and Child’s Health, University of Padova, Padova, Italy; 4 Department of Biology, University of Padova, Padova, Italy; 5 Department of Dental Materials and Biomaterials Research, RWTH Aachen University Hospital, Aachen, Germany

**Keywords:** Autophagy, Chloroquine, Neuroblastoma, Drug resistance, Tyrosine kinase inhibitors

## Abstract

**Background:**

Despite reported advances, acquired resistance to tyrosine kinase inhibitors still represents a serious problem in successful cancer treatment. Among this class of drugs, ponatinib (PON) has been shown to have notable long-term efficacy, although its cytotoxicity might be hampered by autophagy. In this study, we examined the likelihood of PON resistance evolution in neuroblastoma and assessed the extent to which autophagy might provide survival advantages to tumor cells.

**Methods:**

The effects of PON in inducing autophagy were determined both in vitro, using SK-N-BE(2), SH-SY5Y, and IMR-32 human neuroblastoma cell lines, and in vivo, using zebrafish and mouse models. Single and combined treatments with chloroquine (CQ)—a blocking agent of lysosomal metabolism and autophagic flux—and PON were conducted, and the effects on cell viability were determined using metabolic and immunohistochemical assays. The activation of the autophagic flux was analyzed through immunoblot and protein arrays, immunofluorescence, and transmission electron microscopy. Combination therapy with PON and CQ was tested in a clinically relevant neuroblastoma mouse model.

**Results:**

Our results confirm that, in neuroblastoma cells and wild-type zebrafish embryos, PON induces the accumulation of autophagy vesicles—a sign of autophagy activation. Inhibition of autophagic flux by CQ restores the cytotoxic potential of PON, thus attributing to autophagy a cytoprotective nature. In mice, the use of CQ as adjuvant therapy significantly improves the anti-tumor effects obtained by PON, leading to ulterior reduction of tumor masses.

**Conclusions:**

Together, these findings support the importance of autophagy monitoring in the treatment protocols that foresee PON administration, as this may predict drug resistance acquisition. The findings also establish the potential for combined use of CQ and PON, paving the way for their consideration in upcoming treatment protocols against neuroblastoma.

## Background

The third-generation tyrosine kinase inhibitor (TKi) ponatinib (PON) has produced improvements in the treatment of adult patients with chronic myeloid leukemia (CML) [1]. With respect to the first and second generations of BCR-ABL tyrosine kinase protein inhibitors, PON was more successful in eliminating both BCR-ABL wild-type and mutant (BCR-ABL^T315I^) CML cells, thus reducing the possible evolution of resistance due to drug exclusion [2]. Since then, PON has been tested in adolescent patients with CML and pediatric patients with leukemia, with encouraging results [3–5]. Due to its multiple targets, further evaluations have demonstrated the efficacy of PON in affecting other important tyrosine kinases, including EGFR, FGFR, PDGFR, and VEGFR, which are aberrantly activated in different malignancies [6]. The mechanisms of action of PON include the regulation of several intracellular signaling pathways, such as STAT3, PI3K/AKT, and ERK, which are all involved in supporting tumor cell proliferation and survival [7]. However, some studies have found an increased rate of resistance to PON in either pre-clinical or clinical settings [8–10], and a similar finding may therefore be possible in neuroblastoma, in which the efficacy of PON emerged from both *in vitro* and *in vivo* pre-clinical assessments [11, 12]. In a previous high-throughput screening (HTS) study, among 349 compounds tested, PON gave the best results in impeding the growth of neuroblastoma cells [13].

Neuroblastoma is the most common extracranial malignancy, which preferentially occurs in pre-school children. It shows a wide-ranging clinical, histological, and biological heterogeneity and manifests as a localized or metastatic disease [14]. Together, these characteristics determine neuroblastoma tumor staging and patient stratification. At diagnosis, patients with neuroblastoma can be classified into very low, low, intermediate, or high risk groups [15]. The therapy regime is determined by this stratification and is particularly aggressive for the high risk patients, who receive multimodal therapy, autologous stem cell transplantation, and immunomodulatory and pro-differentiation therapy [16]. Nevertheless, these patients rarely achieve complete long-term clinical remission and often face disease recurrence due to the acquired resistance to therapy [17].

In recent years, autophagy has emerged as an important cytoprotective mechanism that is triggered in response to many chemotherapeutic agents. Indeed, several studies have shown the relevance of autophagy in allowing the survival of cancer cells upon the use of anti-neoplastic drugs [18]. In particular, different types of tumor cells exposed to TKi engage autophagy in response to chemical insults in order to relieve cellular stress, and the same behavior has been described in neuroblastoma cells [19, 20].

Autophagy is an evolutionarily conserved mechanism required for proper cell function [21], although it has been connected with tumorigenesis and drug resistance phenomena in a subset of tumor cells [22]. In malignant tissues, autophagy is often associated with the development of secondary (acquired) drug resistance [23]. Despite these roles, several studies have evidenced that autophagy may also function as a tumor promoter [24]. The molecular background defining the balance between cell survival and cell death is determined by the strict connection between autophagy and apoptosis that properly maintains tissue homeostasis [25]. The link between these two processes is complex, and it is not yet completely understood. One of the cross talks between autophagy and apoptosis is determined by the BCL2/Beclin 1 interaction. Under normal conditions, BCL2 prevents Beclin 1 from triggering autophagy, but, upon stress, Beclin 1 is released, allowing its initiation [26]. Different chemotherapy drugs act as stress stimuli when added to tumor cells; hence, unraveling the extent to which autophagy can pilot cancer cell death upon administration of newly proposed anti-neoplastic therapies may be essential for shaping the development of future treatment protocols.

In this study, we assessed the likelihood of autophagy-dependent cytoprotection in neuroblastoma cells during treatment with PON, a third-generation TKi. We evaluated, both *in vitro* and *in vivo,* the synergy between PON and the lysosomal catabolism inhibitor chloroquine (CQ), noting the remarkable effectiveness of this combination treatment in impeding neuroblastoma cell survival and tumor growth.

## Methods

### Cell lines and reagents

The neuroblastoma cell line SH-SY5Y was purchased from DSMZ (Braunschweig, Germany), while SK-N-BE(2) and IMR-32 cells were obtained from ATCC (Manassas, VA). The cells were maintained in RPMI medium supplemented with 10% fetal bovine serum (FBS, Sigma-Aldrich), 1% antibiotics, and glutamine (Gibco). The cells were then exposed to PON (Sigma-Aldrich), CQ (Sigma-Aldrich), or a combination of PON and CQ (COMBO) for the indicated times and at the specified doses. The genetic background of the cell lines is summarized in Supplementary Table S1. *In vivo* studies were done with the less toxic analog hydroxychloroquine (HCQ; Sigma-Aldrich) [27]. Torin 1 (Sigma-Aldrich) was dissolved in DMSO before use, and the cell cultures were regularly tested for the presence of mycoplasmas by PCR. Human cell line authentication was done at BMR Genomics S.r.l. (Padova, Italy).

### Orthotopic neuroblastoma mouse model

Female athymic Nude-Foxn1^*nu*^ mice were purchased from Envigo (Bresso, Italy) and housed under pathogen-free conditions. All the experiments were approved by the ethical committee of the Italian Ministry of Health (n: 661/2016-PR) in compliance with the ARRIVE guidelines (Animal Research: Reporting of In Vivo Experiments). Five-week-old mice were anesthetized with a xylazine–ketamine mix (Xilor 2% plus Imalgene 1000, Merial SpA, Italy), subjected to laparotomy, and inoculated with 1x10^6^ IMR-32 cell line into the left adrenal gland capsule, as previously described [28, 29].

### Metabolic activity assay

To measure the inhibitory concentration of PON that causes 50% cell viability reduction (IC50), 5x10^3^ cells were seeded in a 96-well plate 24h prior treatment to ensure exponential growth. The cells were automatically counted with the Trypan blue exclusion assay (Countess™ cell counter, Invitrogen). Cell viability was assessed for 24h after drug exposure by means of their metabolic activity using 3-(4,5-dimethyl-2-thiazolyl)-2,5-diphenyl-2H-tetrazolium bromide (MTT, Sigma-Aldrich) [30]. The results were compared to control samples treated with an equivalent amount of DMSO.

### Immunoblot analysis, phospho-kinase array, and autophagy array

Protein levels were analyzed as previously described [31]. Briefly, 20 μg of proteins was loaded for each sample on precast 4–20% gradient SDS-PAGE gels (BioRad), transferred to nitrocellulose membrane, and probed with the following primary antibodies: LC3 (Novus Biologicals; 1:1000), p62 (Cell Signaling; 1:500), BECLIN 1 (Novus Biologicals; 1:500), PARP (Cell Signaling; 1:1000), BCL2 (Cell Signaling; 1:1000), VINCULIN (SC Biotechnology; 1:2000), CASPASE 3 (Cell Signaling; 1:200), CASPASE 8 (Cell Signaling; 1:200), ERK total (Cell Signaling; 1:1000), and phospho-ERK (Cell Signaling; 1:1000). The phospho-kinase array was performed as described previously [30], and a human autophagy array (RayBiotech) was performed using 500 μg of total protein extracts, following the manufacturer’s instructions. Protein quantification and signal detection for each assay were performed as previously described [31]. Arrays were analyzed using the ImageJ protein array plugin [32]. The data is presented as fold change relative to the controls.

### Immunofluorescent antibody staining and autophagy flux detection

Immunofluorescence analyses were used for autophagosome and autolysosome detection upon staining with LC3 primary antibody (Novus Biologicals; 1:100) and LAMP-2 (Flarebio Biotech LLC; 1:200), respectively, overnight at 4°C. For *in vitro* drug treatment, neuroblastoma cells were exposed to DMSO (control condition), PON (1 μM for SH-SY5Y and IMR-32, 2 μM for SK-N-BE(2)), CQ (25 μM), and COMBO for 24h. The cells were then fixed with 4% paraformaldehyde (PFA, Sigma-Aldrich) for 15 min and permeabilized with 0.25% Triton X-100 in 3% BSA solution for 10 min. Alexa Fluor 488 (Thermo Fisher; 1:1000) was used as a secondary antibody. Autophagy flux was analyzed using a Premo™ Autophagy Tandem Sensor RFP-GFP-LC3B Kit (Thermo Fisher) according to manufacturer recommendations. Upon autophagy induction, the autophagosomes become double positive (both, GFP and RFP resulting in merged, yellow, signal). Once the lysosome has fused, the pH drops and quenches the GFP, making autolysosomes labeled in red (RFP). The nuclei were stained with Hoechst (Thermo Fisher), and images were acquired with a Zeiss LSM 800 confocal microscope and quantified using Fiji software.

### In vitro drug combination studies

The cytotoxic activity of PON, alone or in combination with CQ, was compared after 72h of treatment using the MTT colorimetric assay. To that end, 5x10^3^ cells were seeded in a 96-well plate the day before the treatment to ensure cell adhesion and growth. For COMBO treatment, the cells were pre-treated for 6h with 25 μM CQ and then treated with the indicated doses of PON or DMSO (control condition). The percentage of cell viability was normalized to the values obtained for the control cells.

### Drug toxicity and autophagy activation in zebrafish embryos

Wild-type (AB/TU) zebrafish embryos were staged and maintained as described previously [33] ; their use was approved by the Italian Ethical Committee OPBA (86/2016-PR)**.** For the *in vivo* drug administration, 48h post-fertilization (hpf) embryos were treated for 12h at 28.5°C with DMSO (control condition), PON (1 μM), CQ (25 μM), and the autophagy inducer Torin 1 (400 nM) as a positive control treatment [34]. The embryos were then fixed in 4% PFA and stained with LC3 antibody (Novus Biologicals; 1:100) as previously described [35]. DAPI (Sigma-Aldrich, 1:10.000) was used for nuclear staining, and images were acquired with a Carl Zeiss Axio microscope and analyzed using Fiji software.

### Efficacy studies and systemic toxicity evaluation in a neuroblastoma mouse model

IMR-32–bearing mice were randomized into *n*=8 per group and evaluated for increased life span and survival. In a second experiment, mice were randomized into *n*=5 per group for tumor growth inhibition and systemic toxicity evaluations. Treatments started 12 days after tumor cell implantation. HCQ (60 mg/kg [36];) and PON (30 mg/kg [13];)—as single agents or in combination (COMBO)—were administered i.p. and by gavage, respectively, every day for 16 days total. In the combination setting, HCQ was administered 20 min before PON. In each experiment, a group of control mice received vehicle only. All animals received the entire schedule of treatment without any sign of systemic toxicity. They were monitored two to three times weekly and euthanized humanely just before showing signs of illness/suffering, such as paraplegia, dehydration, severe weight loss (>15%), or abdominal dilatation.

In the systemic toxicity experiment, the mice were anesthetized with xylazine 24h after the last day of treatment, and blood was collected through the retro-orbital sinus from each mouse into either anticoagulant-free tubes (samples A, for clinical chemistry hepatic, cardiac, and renal evaluations) or K3EDTA coated tubes (samples B, for hematological evaluations). Samples A were centrifuged at 2500×g for 10 min at 4°C, and the levels of serum albumin (ALB), cholinesterase (CHE), glutamic-pyruvic transaminase (ALT), glutamic oxaloacetic transaminase (AST), creatine phosphokinase (CK), and creatinine (CREA) were quantified. Levels of red blood cells (RBC), hemoglobin (HGB), hematocrit (HCT), mean cell volume (MCV), mean cell hemoglobin (MCH), mean cell hemoglobin concentration (MCHC), platelets (PLT), and white blood cells (WBC) were quantified in samples B. All the reported evaluations were performed at the Mouse Clinic, IRCCS Ospedale San Raffaele (Milan). The mice were finally sacrificed, and the tumors were weighed, recovered, fixed in formalin, and embedded in paraffin for subsequent immunohistochemical analysis.

### Histological and immunohistochemical analyses

The paraffin sections were sliced to obtain tumor sections of 5 μm thickness, which were stained with hematoxylin and eosin (H&E) using standard lab protocols. Tumor slices were subjected to immunohistochemical analysis with the following antibodies: LC3 (Novus Biological; 1:200), CD56 (SC Biotechnology; 1:100), active CASPASE 3 (R&D Systems, 1:100), and Ki67 (Dako; 1:200). The sections were incubated with the antibodies overnight at 4°C after blocking the endogenous peroxidase activity with 3% hydrogen peroxide in methanol for 20 min at room temperature and antigen retrieval in citric acid pH 6.0 for 20 min at 90°C. Vectastain ABC horseradish peroxidase anti-rabbit or anti-mouse detection kit (Vector Laboratories) was applied for 30 min. The sections were incubated with diaminobenzidine substrate (Vector Laboratories) to visualize immunoreactivity.

### TUNEL assay

Terminal deoxynucleotidyl transferase dUTP-mediated nick-end labeling (TUNEL) analysis was performed on the mouse tissues using the Click-iT® TUNEL Alexa Fluor® Imaging Assay (Invitrogen), essentially as described by the manufacturer. Briefly, paraffin-embedded tissue sections were de-waxed and incubated with a buffer containing fluorescent nucleotides and the terminal deoxynucleotidyl transferase enzyme for one hour at 37°C. After being washed in PBS, slides were mounted using 80% glycerol. Hoechst (Thermo Fisher) was used to counterstain all the nuclei, and TUNEL-positive signals were determined by counting five randomly selected fields using a Carl Zeiss Axio microscope.

### Transmission electron microscopy (TEM)

Samples were fixed with 2.5% glutaraldehyde (Sigma-Aldrich) in 0.1 M sodium cacodylate buffer pH 7.4 overnight at 4°C. The samples were post-fixed with 1% osmium tetroxide in 0.1 M sodium cacodylate buffer for 1h at 4°C. After three washes with water, the samples were dehydrated in graded ethanol series and embedded in an epoxy resin (Sigma-Aldrich). Ultrathin sections (60–70 nm) were obtained with an Ultrotome V (LKB) ultramicrotome, counterstained with uranyl acetate and lead citrate, and viewed with a Tecnai G2 (FEI) transmission electron microscope operating at 100 kV. Images were captured with a Veleta (Olympus Soft Imaging System) digital camera.

### Statistics

All *in vitro* experiments were performed in triplicate, and the data are presented as mean value ± standard error (SEM). Statistical analyses and graphs were performed using GraphPad Prism 8 (GraphPad, La Jolla, CA). Statistical significance was assessed by one-way analysis of variance (ANOVA) followed by a post hoc Dunnett’s test and a two-sided Student’s t-test.

The *in vivo* data are expressed as mean ± standard deviation (SD). The analyses were performed with GraphPad Prism 5 software—one-way ANOVA with Tukey’s multiple comparison test was used to evaluate differences within treatments, survival curves were drawn as Kaplan–Meier cumulative proportion surviving graphs, and corresponding p-values were calculated using the log-rank (Mantel–Cox) test. *p*<0.05 (95% confidence interval) was considered statistically significant, and significance is indicated as *p<0.05, **p<0.01, or ***p<0.001.

## Results

### Ponatinib triggers autophagy in human neuroblastoma cells

**Fig. 1:**
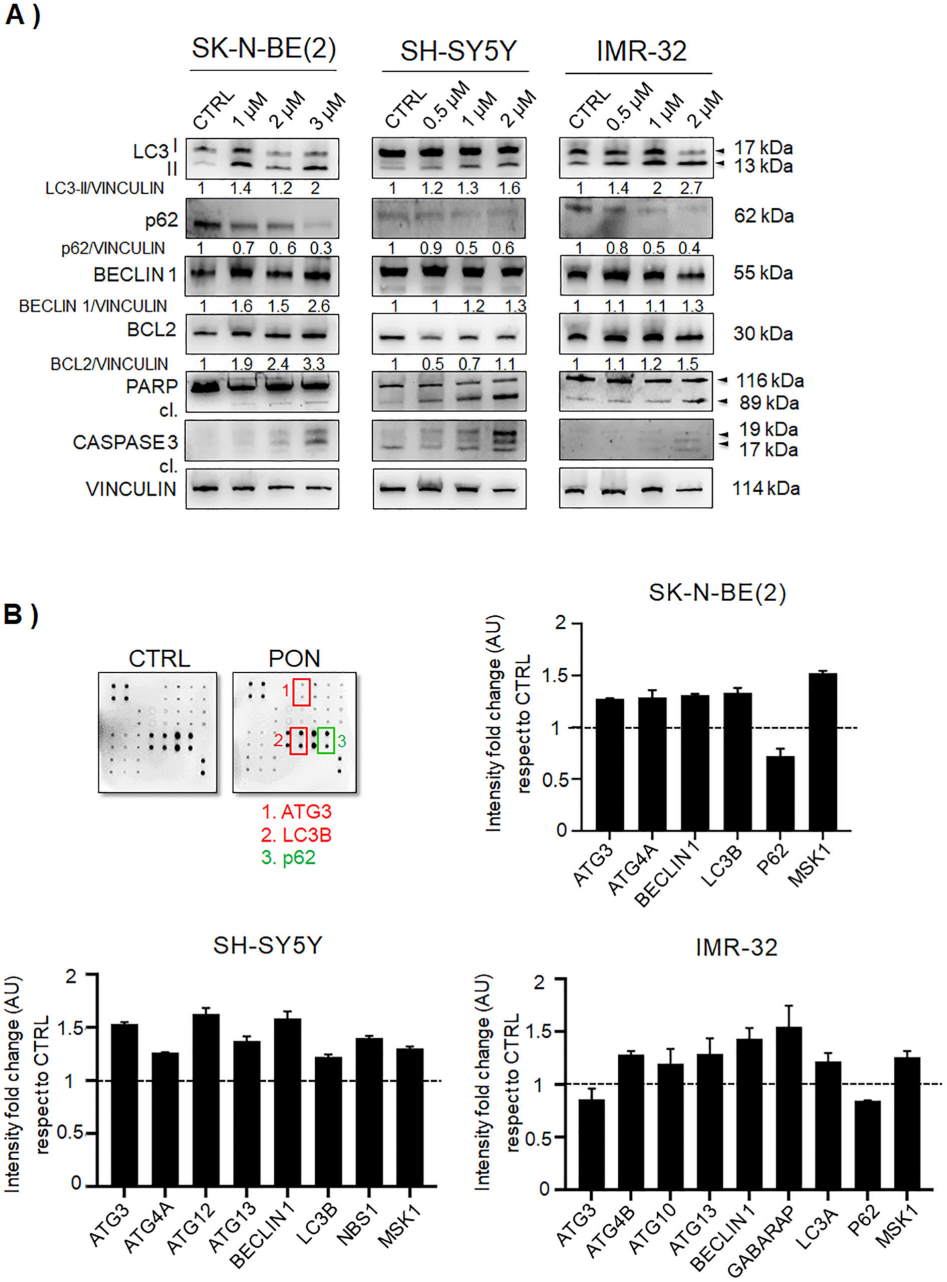
PON induces autophagy in neuroblastoma cells. **a** SK-N-BE(2), SH-SY5Y, and IMR-32 neuroblastoma cell lines were treated with the indicated increasing concentrations of PON or drug vehicle controls (CTRL) for 24 h. Total cell proteins were used for immunoblot analysis of the main autophagy regulators LC3, p62, and BECLIN 1. Apoptotic protein markers BCL2, total and cleaved (cl.) PARP, and cleaved (cl.) CASPASE 3 proteins were also examined. VINCULIN was used as loading control protein. The molecular weights are indicated in kilodaltons (kDa). Numbers indicate each protein/VINCULIN ratio as a fold change with respect to the controls (equal to 1) from two independent immunoblots. **b** Autophagy array was used to delineate the main autophagy players affected by PON in the SK-N-BE(2), SH-SY5Y, and IMR-32 cell lines. The results are normalized to internal positive controls, and the fold change is calculated with respect to the control lysates. ATG3, BECLIN 1, LC3, and MSK1 expressional changes were found in all three cell lines, while the other players differed between the examined cell lines. The data are presented as the value of the mean intensity fold change (AU). The proteins resulting in at least a 20% change are presented (*p* < 0.05)

In a previously established HTS assay among 349 screened small molecule inhibitors, PON was identified as the most promising candidate for abrogating neuroblastoma growth [13]. Here, we assessed a possible functional association between PON and the modulation of autophagy in human neuroblastoma. To this end, three neuroblastoma cell lines—SK-N-BE(2), SH-SY5Y, and IMR-32—were cultured in the presence of increasing concentrations of PON (0.325–10 μM) for 24h to assess the sub-toxic doses (<IC_50_) of the drug (Supplementary Fig. S1A). In all cell lines, PON activity was observed in the μM range, even though the IMR-32 and SH-SY5Y cells were slightly more sensitive to PON than SK-N-BE(2) (Supplementary Fig. S1B). To determine the extent to which sub-toxic doses of PON affect autophagy and apoptosis, we examined the levels of the main regulators of each of these two processes. PON significantly decreased the levels of polyubiquitin-binding cargo protein p62 (SQSTM1) and provoked the accumulation of LC3-II in a dose-dependent manner, implying the activation of the autophagic flux (Fig. 1a). The level of total Beclin 1 protein showed a modest increase (Fig. 1a), and the crosstalk between autophagy and apoptotic cell death [25] was followed by means of BCL2 protein levels, PARP, and CASPASE 3 protein cleavage. These results confirmed a cell type–dependent modulation of BCL2 (anti-apoptotic protein) levels, with the most evident decrease in the SH-SY5Y cells (Fig. 1a). The cleavage of PARP protein and CASPASE 3 were evident for the IC50 doses of PON; more importantly, the activation of autophagy was already observed at the sub-lethal doses of drugs, while the pro-apoptotic signals were visible at doses approaching the IC50 of PON (Fig. 1a). Besides LC3 and p62, other autophagy-related proteins participate in autophagosome formation [37]. As shown in Fig. 1b, treatment with PON significantly modulated the expression of additional autophagy marker proteins, including ATG3, ATG4, and MSK1 (Fig. 1b). These results confirm that sub-toxic doses of PON induce autophagy in neuroblastoma cells and that autophagy anticipates apoptotic cell death triggered by the drug treatment.

### Ponatinib affects the phosphorylation of different protein kinases

Several pathways have been reported as being affected by PON [13, 38]. To assess how the pathway profiling differs in the three neuroblastoma cell lines treated with sub-IC_50_ PON, we performed a phospho-kinase array (Supplementary Fig. S2A, left panel). We identified 16 out of 39 (41%) kinase phosphorylation sites (downregulated: CREB^S133^, EGFR^Y1086^, ERK1/2^T202/Y204, T185/Y187^, HSP27^S78/S82^, LCK^Y394^, LYN^Y397^, MSK1/2^S376/S360^, P38A^T180/Y182^, SRC^Y419^, WNK1^T60^, YES^Y426^, STAT3^S727^, PDGFRβ^Y751^, and β-CATENIN total protein; upregulated: CHK-2^T68^, P53^S392^) with changed levels in at least two different cell lines. The expression in these samples varied more than 25% with respect to the corresponding controls (data not shown). Seven phospho-kinases (44%) were downregulated in all three cell lines and formed a protein–protein interaction network (Supplementary Fig. S2B). Moreover, pathway enrichment analysis (www.pathwaycommons.org) determined the involvement of these kinases in the regulation of PI3K/AKT signaling. The remaining nine modifications (56%) had the same phosphorylation changes in two out of three cell lines. In particular, ERK1/2 was found to be downregulated in the SK-N-BE(2) and IMR-32 cells but upregulated in the SH-SY5Y cells (Supplementary Fig. S2A, right panel), implying a possible MYCN-dependent response to PON [39].

### Ponatinib primes the formation of autophagic vesicles in human neuroblastoma cells

**Fig. 2:**
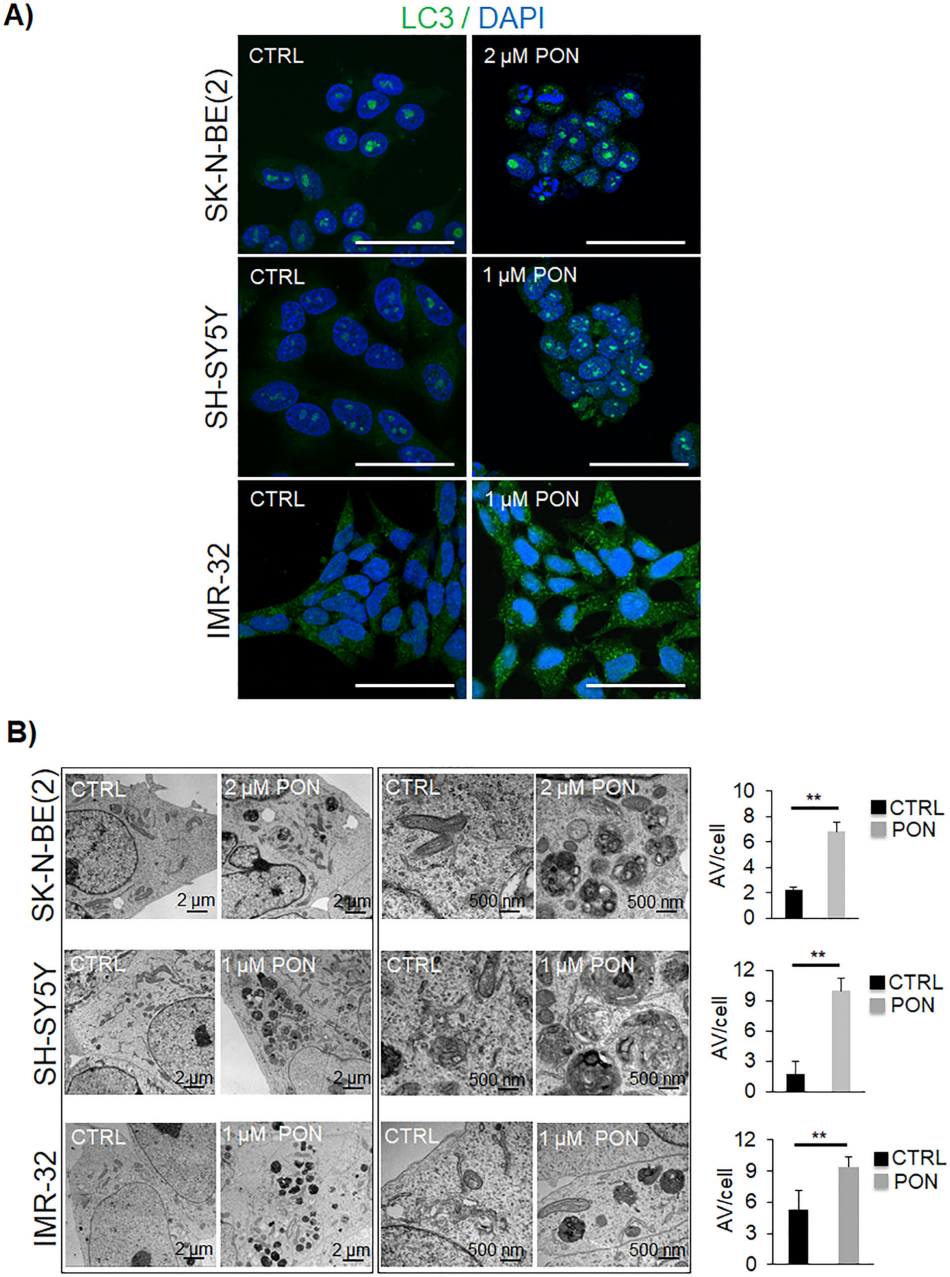
PON promotes autophagy vesicle accumulation in human neuroblastoma cells*.* **a** Tumor cells were treated with vehicle (CTRL) or PON at the indicated concentrations for 24 h. The presence of cytosolic puncta was detected through immunofluorescence analyses with LC3 antibody (green). Nuclei were counterstained with DAPI (blue). Scale bar, 50 μm. **b** Transmission electron microscopy images present the autophagic vacuoles (AV) in SK-N-BE(2), SH-SY5Y, and IMR-32 cells after 24 h of treatment with vehicle (CTRL) or PON. The scale bar is indicated in each image. Histograms on the right show the mean number of AVs formed per cell. **p < 0.01

To further confirm the activation of the autophagic flux in neuroblastoma cells treated with PON, we performed immunofluorescence staining. Using immunocytochemistry, we detected the accumulation of LC3-positive vacuoles in treated cells (Fig. 2a). More precisely, PON markedly increased cytoplasmic LC3 puncta formation in all three neuroblastoma cell lines with respect to the DMSO-treated control cells (Fig. 2a). TEM experiments confirmed the accumulation of autophagic vesicles (Fig. 2b); together, these results corroborate that PON triggers autophagy in neuroblastoma cells.

### Autophagy inhibition sensitizes neuroblastoma cells to treatment with ponatinib

**Fig. 3:**
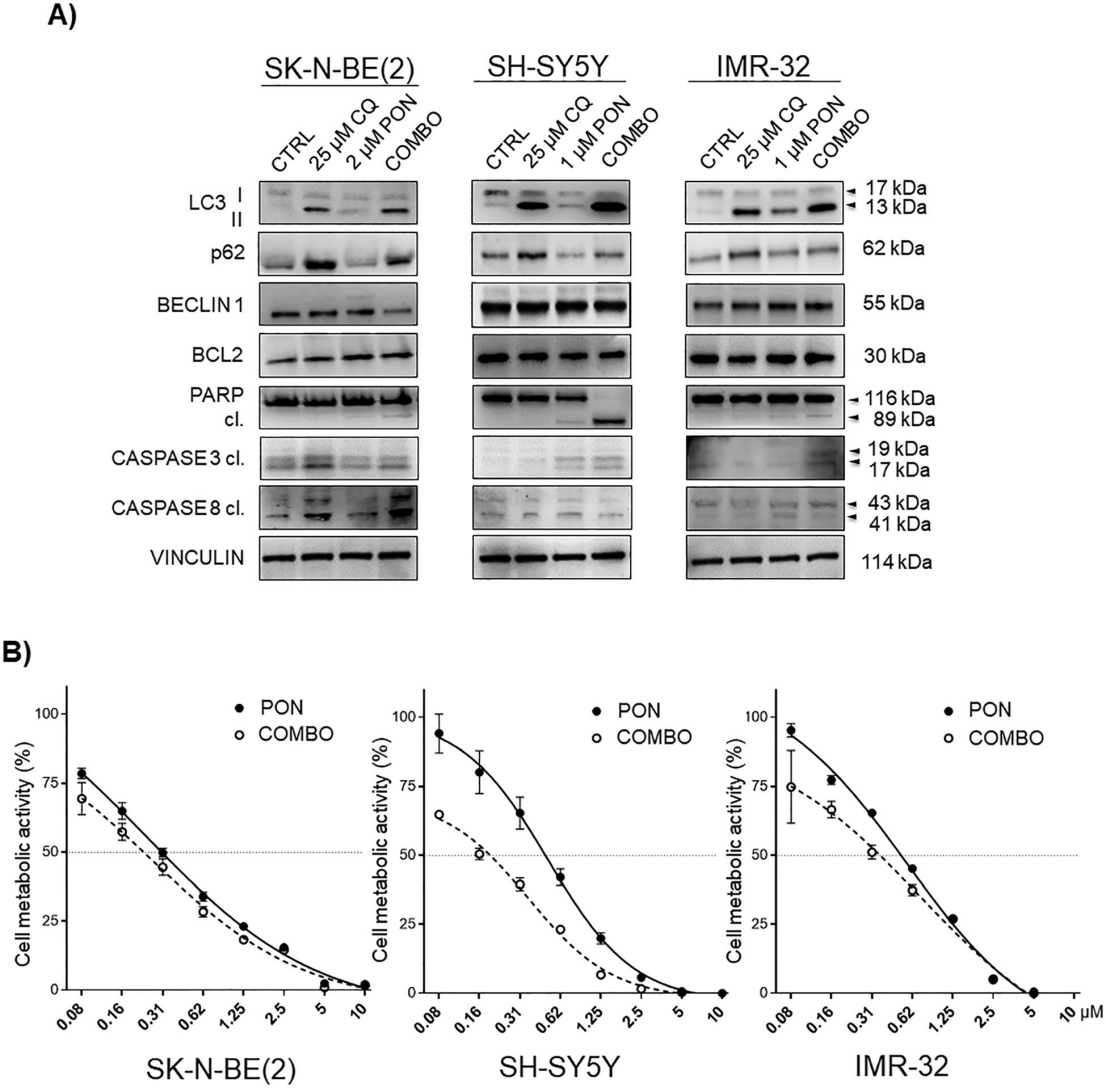
CQ interrupts PON-induced autophagy and sensitizes neuroblastoma cells to PON-dependent cytotoxicity. **a** Neuroblastoma cells were pre-treated with CQ for 1 h, and PON was then added for 24 h at the indicated concentrations. Total protein lysates were used for the immunoblotting analysis of the main autophagy regulators p62, LC3, and BECLIN 1. Apoptosis was assessed by means of BCL2, total and cleaved (cl.) PARP, cleaved (cl.) CASPASE 3, and CASPASE 8 proteins. VINCULIN was used as a loading control protein. The molecular weights are indicated in kilodalton (kDa). **b** Cell metabolic activity assay (MTT) was done in neuroblastoma cells pre-treated with 25 μM of CQ, treated with PON alone, or co-treated (COMBO) with the indicated doses (μM) of drugs for 72 h. Data are normalized to the control samples (100%). Symbols and bars represent the mean ± SEM of three independent experiments

To assess whether autophagy triggered by PON could have a cytoprotective effect in neuroblastoma cells, we combined PON with CQ [40]. Increased p62 levels and accumulation of LC3-II in the samples treated with CQ alone suggested a defective autophagic flux (Fig. 3a). Instead, the parallel increase in LC3-II and decrease in p62 levels observed upon treatment with PON alone confirmed that the treatment itself enhanced the autophagic flux (Fig. 3a). Consistently, single treatments with PON or CQ caused a marked accumulation of green signal—a sign of autophagy vesicles accumulation. COMBO-treated cells displayed a reduced amount of LC3-positive green puncta with respect to the single treatments (Supplementary Fig. S3); more importantly, COMBO treatment potentiated the PON-induced toxicity, as revealed by increased PARP and CASPASE 3 cleavage (Fig. 3a) and the appearance of fragmented nuclei (Supplementary Fig. S3). These results confirm that the observed autophagy had a cytoprotective role in neuroblastoma cells and imply that its inhibition can be used to increase tumor cells’ sensitivity to PON. However, both treatments produced only slight changes in the levels of total BCL2 and BECLIN1 proteins (Fig. 3a), indicating that they cannot be used confidently as markers for immediate autophagy evaluation in neuroblastoma cells when only a single temporal point is available for testing. The metabolic activity of neuroblastoma cells was significantly more attenuated upon COMBO treatment, especially at the lower PON concentrations (Fig. 3b), indicating that the introduction of CQ had a pro-synergistic nature, particularly in the SH-SY5Y and IMR-32 cells. However, the synergistic effect of CQ was less evident in the SK-N-BE(2) cells, which showed an important sensitivity to CQ treatment alone, as confirmed by intense cleavage of not only CASPASE 3, but also CASPASE 8. The latter result implies that death receptors induced apoptosis [41] in the SK-N-BE(2) cells only upon addition of CQ, but not PON. Taken together, these results show that neuroblastoma cells activate autophagy as a pro-survival cue in response to PON exposure and imply that the greatest efficacy in impeding cancer cell survival comes from lowered doses in a combination treatment.

### Combination therapy impedes the formation of autolysosomes in human neuroblastoma cells

**Fig. 4:**
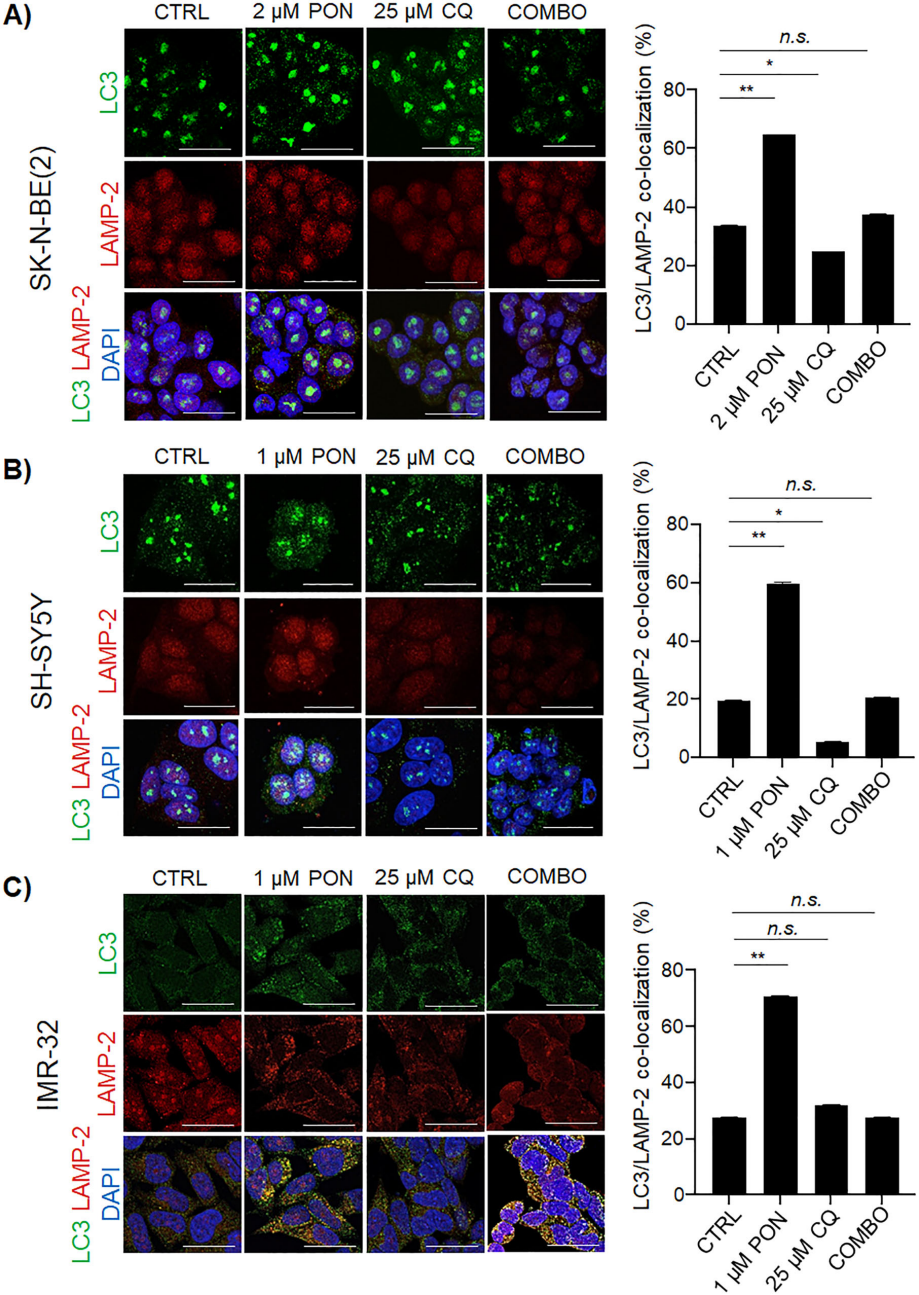
Combo therapy interferes with autophagy flux in human neuroblastoma cells. **a-c** Representative co-immunostaining of LC3 (green) and LAMP-2 (red) proteins are shown for the neuroblastoma cells treated according to the indicated schemes. Histograms show the percentage (%) of double-positive (yellow puncta) staining (autolysosomes). DAPI was used to counterstain nuclei. Scale bar, 100 μm. *p < 0.05; **p < 0.01; *n.s.* – not significant

By performing co-immunostaining for LC3 (green) and LAMP-2 (red) we revealed high amounts of enlarged lysosomes in PON-treated neuroblastoma cells, whereas in DMSO-treated (control condition) cell cultures, lysosomes appeared as smaller punctuate structures (Fig. 4). Enlarged lysosomes were only detectable after PON administration, primarily in IMR-32 cells. Moreover, in the control condition cells, the co-localization of autophagosomes with lysosomes into autolysosomes (fusion events) showed that autophagy is already active at the basal levels in neuroblastoma cells in the absence of a drug, whereas it was significantly enhanced during treatment with PON. Indeed, PON-treated cells showed a significantly enhanced LC3/LAMP-2 co-localization (Fig. 4 a-c), indicating a higher rate of autophagosome–lysosome fusion. As expected, in CQ-treated cultures, co-localization of LC3 and LAMP-2 was comparable to (Fig. 4 a, c) or less than (Fig. 4b) the controls, confirming that CQ acts as a late-stage autophagy inhibitor by impeding lysosomes (red puncta) from fusing to autophagosomes (green puncta). The results were further confirmed using a RFP-GFP-LC3B tandem fluorescent construct that allows an enhanced dissection of the maturation of autophagosomes into autolysosomes (Supplementary Fig. S4A-C). Increase of both, green (GFP) and red (RFP) fluorescence intensity in PON treated cells versus their controls sustained that PON induced autophagy flux. After CQ treatment, the accumulation of yellow (merged GFP-RFP signal) foci was visible confirming the efficacy of CQ treatment in impeding creation of autolysosomes and hence GFP-quenching. The COMBO treatment blocked PON-dependent autophagy and autophagy flux as sustained by the reduced number of the GFP and RFP dots when compared to PON treated samples (Supplementary Fig. S4A-C). Taken together, these approaches evidence that COMBO treatment could be sufficient to inhibit the protective role of autophagy while enhancing neuroblastoma cells’ vulnerability to PON. Analysis of all neuroblastoma cell lines exposed to PON provided similar results (Fig. 4 a-c and Supplementary Fig. 4A-C), highlighting the autophagy activation as a common cytoprotective mechanism against this drug.

### PON treatment activates autophagy in zebrafish embryos

To answer the question of whether PON may induce autophagy *in vivo*, we first treated wild-type zebrafish embryos with sub-lethal doses. We compared the number of LC3-positive puncta in embryos treated with PON alone or in combination with CQ (Supplementary Fig. S5). Torin 1, a small molecule that induces autophagy activation by inhibiting mTOR complex 1 (mTORC1), was used as a positive control. Treatment with PON provoked a significant increase in LC3-positive (green) puncta with respect to DMSO-treated control embryos. This data confirmed the induction of PON-dependent autophagy *in vivo*. In contrast, treating embryos with CQ alone (25 μM) did not significantly affect the number of LC3-positive events. The COMBO treatment decreased the number of cells that activated autophagy in the presence of PON alone, reaching, in this way, a level of LC3-positive puncta comparable to that found in the DMSO-treated control embryos.

### Combination treatment decreases tumor growth and increases survival in mice

**Fig. 5:**
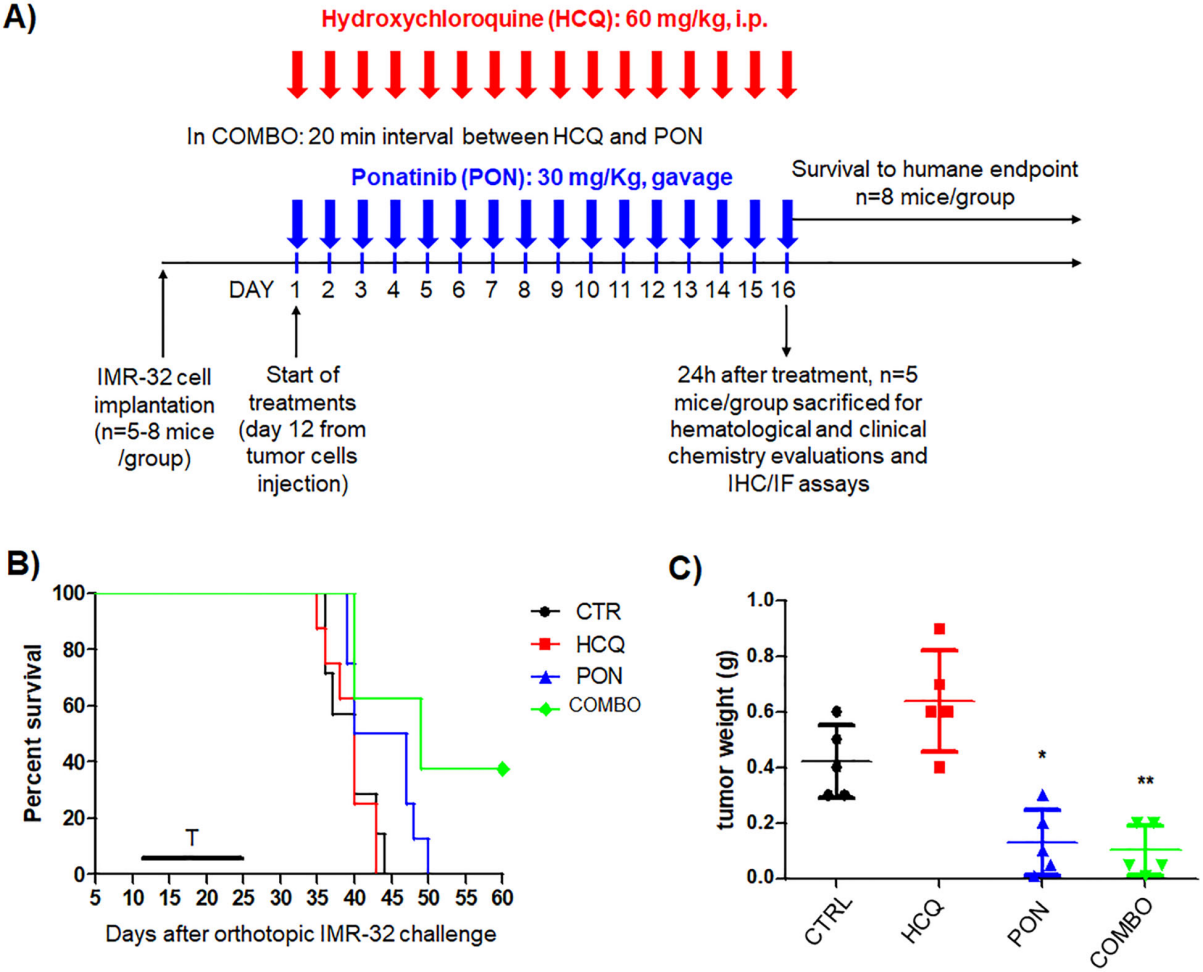
CQ potentiates PON-induced cytotoxicity in the neuroblastoma mice model. **a** The treatment scheme is presented. Mice (*n* = 8 per group) orthotopically injected with IMR-32 cells were treated every day for 16 days, as graphically represented. **b** Treatments (T) started 12 days after tumor cell implantation. Survival: *p < 0.05, PON vs CTRL; **p < 0.01, COMBO vs CTRL; *p < 0.05, COMBO vs PON. **c** IMR-32–bearing mice (*n* = 5 per group) were treated as above and sacrificed 24 h after the last day of treatment. *p < 0.05, PON vs CTRL; **p < 0.01, COMBO vs CTRL

Next, to investigate the effects of a COMBO treatment on neuroblastoma tumors *in vivo*, an orthotopic model was established by inoculating IMR-32 cells into the adrenal gland of athymic nude mice (Fig. 5a). In the first set of experiments, a COMBO treatment strategy was evaluated in terms of the percentage of increased life span (ILS%) and survival. A combination of HCQ with PON led to a 22.5% ILS (median survival: control mice, 40 days; COMBO-treated mice, 49 days) and a significant increase in survival versus both the control (*p*=0.007) and PON administered alone (*p=*0.04) (Fig. 5b). Moreover, in IMR-32 xenografts, HCQ alone did not significantly increase the mice’s life span compared to the controls, while a significant improvement of the anti-tumor effect was obtained in mice treated with COMBO therapy (Fig. 5b). From a mechanistic point of view, in the second set of experiments, the tumors were analyzed 24h after the last drug administration. As shown in Fig. 5c, PON treatment significantly decreased tumor weight in comparison with the control animals, while the COMBO treatment further increased the anti-tumor effects. Notably, treatment with HCQ alone showed a slight, but not significant, increase in tumor weight (Fig. 5c). On the other hand, the reduction in tumor weight in mice treated with PON alone and in the COMBO set was significant in comparison to the control tumor masses (Fig. 5c).

**Fig. 6:**
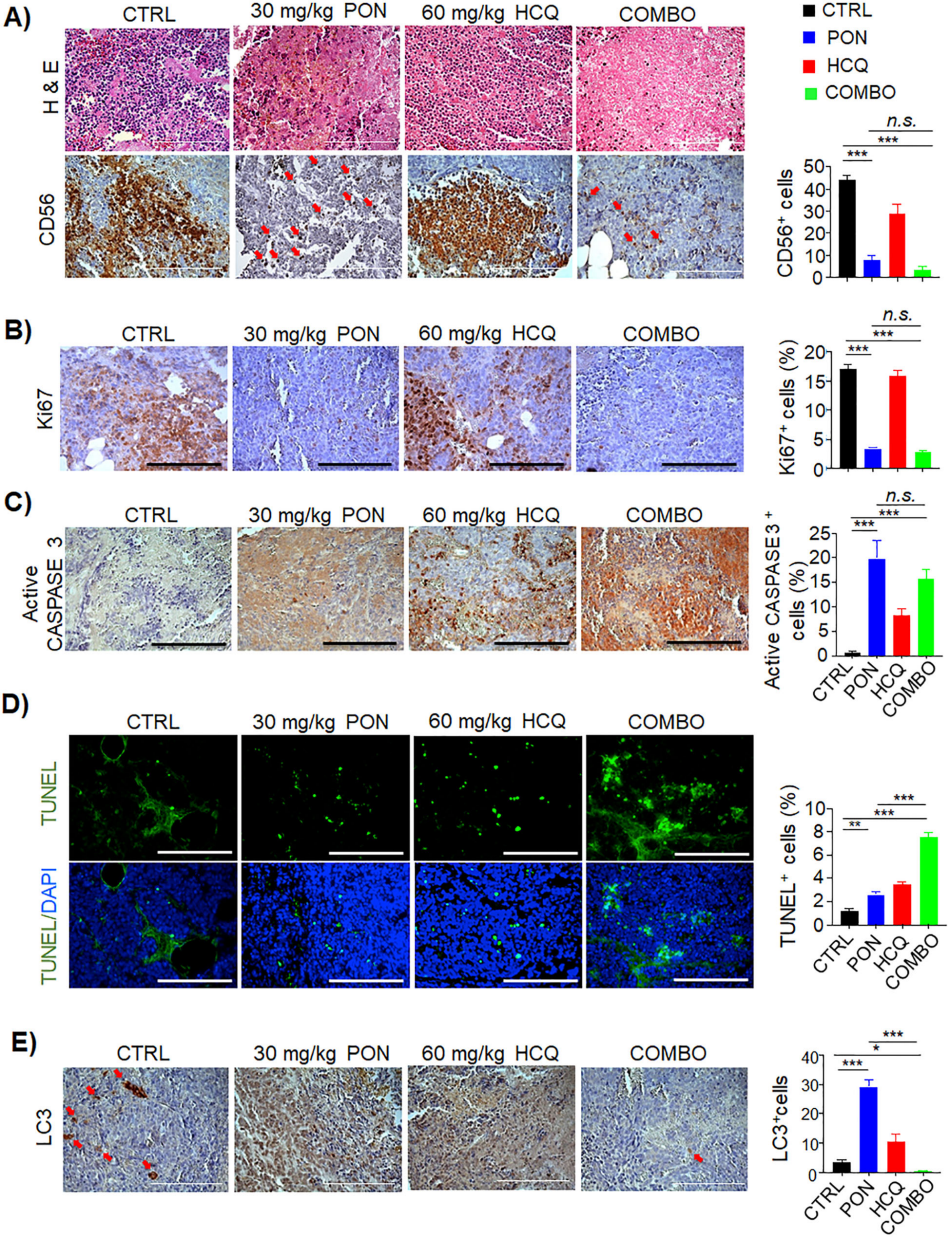
Histopathological examination of post-therapy neuroblastoma tumors confirms the efficacy of combination treatment in vivo. **a** Hematoxylin & Eosin (H&E) of tumor tissues and immunohistochemistry staining of CD56 proteins were carried out. Red arrows indicate positive cellular labeling. Scale bar, 150 μm. **b** Immunohistochemistry staining of Ki67, **c** active CASPASE 3, and **d** TUNEL assay for the detection of proliferating and apoptotic cells, respectively. The percentage (%) of positive events is reported in the histograms on the right. Scale bar, 150 μm. **e** Immunohistochemistry staining of LC3-positive cells in resected post-therapy tumors. Red arrows indicate positive cellular labeling. Scale bar, 150 μm. The percentage (%) of LC3-positive cells is reported in the histogram on the right. *p < 0.05, **p < 0.01, ***p < 0.001

To define the immunohistochemical profile of local tumor cells, we performed histological analyses on paraffin tumor sections. Neuroblastoma tumor masses at the site of inoculation strongly expressed human CD56, confirming their human origin (Fig. 6a and Supplementary Fig. S6). This immunoreactivity was significantly decreased in PON- and COMBO-treated mice (Fig. 6a). To test whether the survival observed upon COMBO treatment was a consequence of impaired tumor growth or increased tumor cell death, we analyzed the proliferation and apoptotic rates in the collected tumor masses. Notably, the slight, but not significant, increase in tumor weight found in HCQ-treated mice (Fig. 5c) was sustained by more intense Ki67 immunoreactivity (Fig. 6b and Supplementary Fig. S6). On the other hand, the reduction in tumor weight in PON- and COMBO-treated mice was a cumulative incidence of the reduction of Ki67-positive (proliferating) cells and the enhancement of active CASPASE 3 and TUNEL-positive (dead) cells (Fig. 6 c-d and Supplementary Fig. S6). To study the role of autophagy in this scenario, the intracellular localization of LC3 was determined by immunohistochemical staining using an anti-LC3 antibody (Fig. 6e and Supplementary Fig. S6). The control animals exhibited a low level of autophagy activation, whereas the PON-treated mice showed an increase in LC3 positivity, confirming the results obtained *in vitro*. Concomitantly, HCQ inhibited autophagy by inducing the accumulation of LC3 in treated mice, whereas COMBO treatment caused a more remarkable reduction in LC3-positive signals (Fig. 6e).

Finally, the proposed treatments were evaluated for eventual toxicity induction. Importantly, no weight loss was evidenced in any of the treated groups (data not shown), and no chronic clinical chemistry and hematological toxicities emerged from the quantification of the level of the corresponding markers in plasma and blood, respectively (Supplementary Fig. S7A-B). Collectively, our data suggests that the inhibition of autophagy by HCQ may further enhance the anti-cancer effects of PON *in vivo* without showing side effects.

## Discussion

Several recent studies have proposed PON—a pan-tyrosine kinase inhibitor—for the treatment of neuroblastoma [11, 42]. In different pre-clinical models of this pediatric cancer, PON was more effective in inhibiting tumor cell growth than several other anti-tumor compounds [12, 13]. However, PON does not affect neuroblastoma cells through the chimeric protein BCR-ABL, since it is not found in this pediatric malignancy, but rather through other TKs, such as FGFR1 or EGFR [12, 38]. The appearance of drug resistance continues to be one of the most critical impediments to cancer therapies that foresee the adoption of TKi.

In the present study, we investigated whether the use of PON, similar to other TKi [9], may correlate with the activation of cytoprotective autophagy, leading eventually toward acquired drug resistance. We therefore assessed, *in vitro* and *in vivo*, the levels of autophagy in neuroblastoma cells treated with sub-toxic doses of PON and subsequently examined the effects of combined treatment with PON and the autophagy inhibitor CQ on neuroblastoma cell survival and tumor growth.

Our data revealed PON-dependent activation of autophagy, both *in vitro* and *in vivo,* thus highlighting this cell process as an active mechanism that is plausibly involved in the development of drug resistance in neuroblastoma. In the analyzed neuroblastoma cell lines, PON caused a rapid increase in the autophagy levels found in control cells. This scenario may be explained as an attempt by tumor cells to rapidly deal with toxic insults inflicted by PON. To monitor autophagic flux, the levels of LC3-II and p62 proteins were analyzed [43]. In neuroblastoma cells treated with PON, the observed changes in both LC3-II and p62 protein levels were indicative of a dose-dependent activation of autophagy, and PON treatment triggered a formation of LC3/LAMP-2-positive autophagic vesicles in the cell cytosol. Co-localization of LC3/LAMP-2 occurs during the final phases of the autophagy process, during which the selected constituents are captured and degraded by lysosomal enzymes within autolysosomes. Detection of LAMP-2 protein levels and positioning allowed us to monitor the autophagosome–lysosome fusion process in PON-treated neuroblastoma cells. This result, along with the data obtained by electron microscopy, immunoblotting and the pH-dependent LC3 color-code changes, gave a definite confirmation that PON caused autophagic flux activation in neuroblastoma cells.

It is not rare for tumor cells to trigger autophagy in order to assure their survival after chemotherapy and radiotherapy treatments [44]. Autophagy-dependent mechanisms of acquired resistance to TKi that involve AXL signaling have recently been reported [45]; these mechanisms are activated after EGFR inhibition, upon which AXL takes over the transduction of the interrupted signaling. The cytoprotective role of AXL has been recognized in different tumor types [46], including non-*MYCN*–amplified neuroblastoma [47]. The AXL targeting in neuroblastoma *in vitro* models appears to be an effective cytotoxic approach in neuroblastoma, without causing significant variations in autophagy levels [30], but further investigations are required to delineate a possible link between changes in the AXL expression and the observed cytoprotective autophagy in PON-treated neuroblastoma cells.

Based on current knowledge, pharmacological inhibitors of autophagy could be an effective adjuvant therapy for enhancing the cytotoxic effects of current chemotherapy protocols. Several autophagy inhibitors have so far been proposed [48]; of these, chloroquine (CQ)—and its analog, hydroxychloroquine (HCQ)—inhibit lysosome fusion to the autophagosome and impair further maturation into degradative autolysosomes. Indeed, they are considered to be late-phase autophagy inhibitors [43, 49]. CQ and HCQ are also effective anti-malaria drugs, having anti-inflammatory cues as well [50], and several clinical trials have reported the possible effectiveness of CQ and HCQ in cancer-related therapies [51]. Neuroblastoma cells were successfully tested for the combined use of PON and either CQ or HCQ; CQ hindered autophagosome and lysosome fusion in PON-treated neuroblastoma cells, thus prompting apoptotic death induction. Moreover, another important outcome of the adopted combination treatment was that lower concentrations of PON were sufficient to induce apoptosis in neuroblastoma cells.

Autophagy is a biochemical process that remains active at a basal level of physiological conditions [37]. We exploited the advantages of the zebrafish *in vivo* model to determine whether PON causes changes in the level of autophagic vesicles with respect to control wild-type embryos. The results confirmed the significant upregulation of LC3-positive puncta in PON-treated embryos with respect to the control group, implying that PON triggers pro-autophagic events. In the neuroblastoma orthotopic mouse model, the reduction in tumor size was potentiated in mice treated with a combination (PON and HCQ) with respect to single (PON) therapy. The combination strategy was associated with the increased expression of active CASPASE 3 and TUNEL-positive cells with respect to a single treatment, without causing any systemic toxicity. These data further corroborate the results obtained *in vitro*, in which the synergistic effects of the proposed combination treatment were determined.

## Conclusions

In summary, we demonstrated that PON activates cytoprotective autophagy in neuroblastoma cells if used as a single treatment. The inhibition of autophagy by compounds already available, such as CQ and HCQ, could help in lowering the doses of PON required to affect tumor cell growth while improving its anti-neoplastic effects. These findings are of great relevance, since significant side effects have been reported in leukemia patients upon treatment with PON [45], and we may therefore need to decrease the concentration of such TKi in therapeutic protocols adopted for childhood malignancies, while maintaining their efficacy. One option could be an adjuvant therapy with CQ. Collectively, our results highlight the potential of the combined action of PON and CQ, giving a preliminary insight into the efficacy of this treatment strategy as a novel targeted therapy approach in neuroblastoma.

## Supplementary information

**Additional file 1: Supplementary Figure S1.** PON impaired the viability of neuroblastoma cells in a concentration-dependent manner. (A) SK-N-BE(2), SH-SY5Y, and IMR-32 neuroblastoma cell lines were treated with increasing concentrations of PON (0.325–10 μM) or drug vehicle (CTRL), and cell metabolic activity was determined by an MTT test. (B) IC_50_ was calculated 24 h post-treatment. The data are presented as percent change calculated with respect to CTRL cells (100%).

**Additional file 2: Supplementary Figure S2.** PON affects the phosphorylation of different protein kinases. (A) Representative image of human phosphorylation-kinase assay (part a and b) performed with total cell lysates of neuroblastoma cells treated with vehicle (CTRL; C) or sub-IC_50_ of PON for 24 h. The levels of ERK1/2 phosphorylation (red squares) were analyzed using western blot (right panel) to confirm the inversed expression in three cell lines. VINCULIN was used as a loading control protein. The molecular weights are indicated in kilodalton (kDa). (B) Interaction of the seven common phospho-kinases modified in PON-treated samples versus their controls was studied using public pathway and interactions databases (www.pathwaycommons.org). Connecting lines include different types of interactions between SCR, PDGFRB, EGFR, YES1, MAPK14 (P38), RPS6KA5 (MSK1/2), and WNK1, containing interactions or modifications, as indicated.

**Additional file 3: Supplementary Figure S3.** Combination approach reduced autophagosome accumulation in human neuroblastoma cells*.* Tumor cells were treated as indicated, and the presence of cytosolic puncta was detected through immunofluorescence analyses performed with an anti-LC3 antibody (green). Nuclei were counterstained with DAPI (blue). Scale bar, 50 μm.

**Additional file 4: Supplementary Figure S4.** PON stimulates autophagy flux. a) SK-N-BE(2), SH-SY5Y, and IMR-32 neuroblastoma cells expressing an RFP-GFP-LC3 construct were treated for 24 h with the indicated compounds. The neutral pH LC3-positive autophagosomes (GFP and RFP positive dots) and the acidic pH LC3-positive autolysosomes (RFP positive dots) were detected with a confocal microscope. The nuclei were counterstained with Hoechst (blue signal). Scale bar, 50 μm. The quantification of GFP and RFP dots is reported in the histograms on the right. The data are presented as the mean number of positive dots per cell ± SEM. *p < 0.05; **p < 0.01; *n.s.* – not significant

**Additional file 5: Supplementary Figure S5.** PON treatment activates autophagy in vivo*.* Representative images show LC3-positive autophagosomes (green puncta) in the trunk area (blue square) of wild-type zebrafish embryos treated as indicated. Nuclei were counterstained with DAPI (blue). A total of 20 embryos was analyzed for each condition per experiment. Scale bars, 50 μm. The data are presented as mean ± SEM of three independent experiments. *p < 0.05; **p < 0.01; *n.s.* – not significant

**Additional file 6: Supplementary Figure S6.** Quality control confirms antibody specificity for immunohistochemical staining. Histopathological examination of tumor sections isolated from nude mice bearing isogenic IMR-32 orthotopic tumors was performed using only the secondary antibodies applied in Fig. 6. Negative controls were primarily used to evaluate the specificity of the immunohistochemistry staining and to identify false-positive staining reactions for each antibody used. Scale bar, 100 μm.

**Additional file 7: Supplementary Figure S7.** Combination treatment exerts no hematological or clinical chemistry toxicities. IMR-32–bearing mice (*n* = 5 per group) were treated as reported in Fig. 5 and sacrificed 24 h after the last day of treatment. (A) Red blood cells (RBC), hemoglobin (HGB), hematocrit (HCT), mean cell volume (MCV), mean cell hemoglobin (MCH), mean cell hemoglobin concentration (MCHC), red blood cell distribution width (RDW-SD), platelets (PLT), and white blood cells (WBC) were analyzed. (B) Serum albumin (ALB), glutamic-pyruvic transaminase (ALT), glutamic oxaloacetic transaminase (AST), cholinesterase (CHE), creatine phosphokinase (CK), and creatinine (CREA) were analyzed.

**Additional file 8:** **Supplementary Table S1.** Genetic background of the neuroblastoma cell lines used in this study.

## Data Availability

Not applicable.

## Abbreviations

CQ: Chloroquine
HCQ: Hydroxychloroquine
ILS: Increased life span
PON: Ponatinib
TKi: Tyrosine kinase inhibitor
TEM: Transmission electron microscopy
