# Supplementary figures and images for: Autophagic flux inhibition enhances cytotoxicity of the receptor tyrosine kinase inhibitor ponatinib
Source: J Exp Clin Cancer Res. 2020 Sep 22;39:195. doi: 10.1186/s13046-020-01692-x (PMC7507635; doi:10.1186/s13046-020-01692-x)

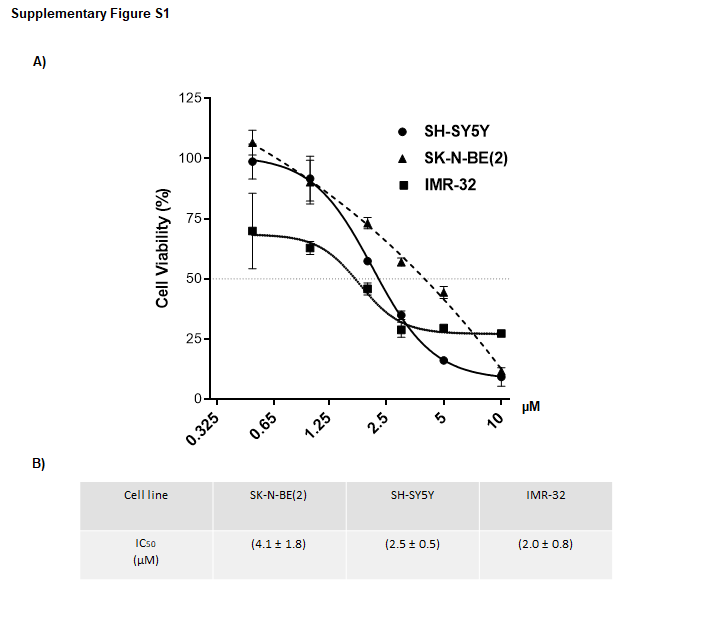

Supplement: Supplementary file 1 — Additional file 1: Supplementary Figure S1. PON impaired the viability of neuroblastoma cells in a concentration-dependent manner. (A) SK-N-BE(2), SH-SY5Y, and IMR-32 neuroblastoma cell lines were treated with increasing concentrations of PON (0.325–10 μM) or drug vehicle (CTRL), and cell metabolic activity was determined by an MTT test. (B) IC50 was calculated 24 h post-treatment. The data are presented as percent change calculated with respect to CTRL cells (100%). [file 13046_2020_1692_MOESM1_ESM.tif]

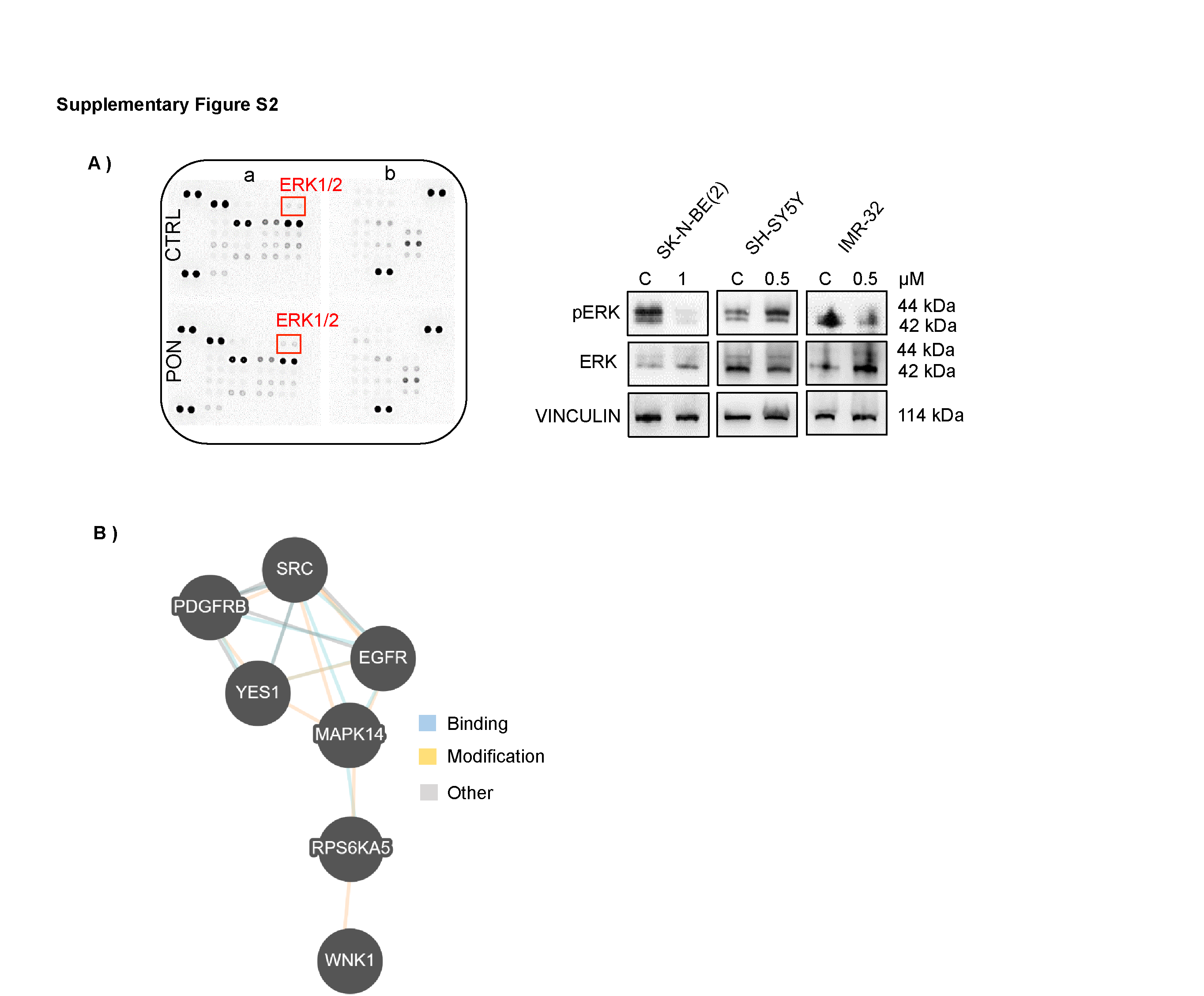

Supplement: Supplementary file 2 — Additional file 2: Supplementary Figure S2. PON affects the phosphorylation of different protein kinases. (A) Representative image of human phosphorylation-kinase assay (part a and b) performed with total cell lysates of neuroblastoma cells treated with vehicle (CTRL; C) or sub-IC50 of PON for 24 h. The levels of ERK1/2 phosphorylation (red squares) were analyzed using western blot (right panel) to confirm the inversed expression in three cell lines. VINCULIN was used as a loading control protein. The molecular weights are indicated in kilodalton (kDa). (B) Interaction of the seven common phospho-kinases modified in PON-treated samples versus their controls was studied using public pathway and interactions databases (www.pathwaycommons.org). Connecting lines include different types of interactions between SCR, PDGFRB, EGFR, YES1, MAPK14 (P38), RPS6KA5 (MSK1/2), and WNK1, containing interactions or modifications, as indicated. [file 13046_2020_1692_MOESM2_ESM.tif]

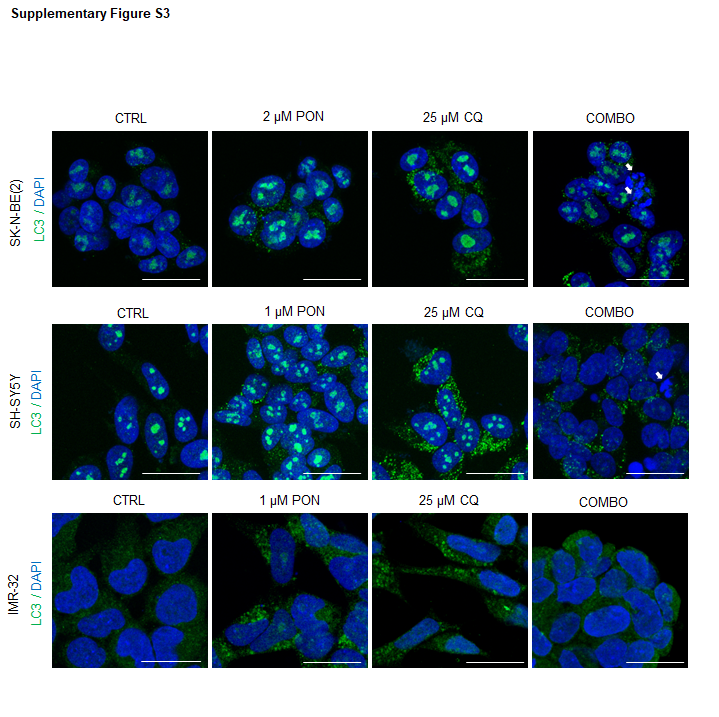

Supplement: Supplementary file 3 — Additional file 3: Supplementary Figure S3. Combination approach reduced autophagosome accumulation in human neuroblastoma cells. Tumor cells were treated as indicated, and the presence of cytosolic puncta was detected through immunofluorescence analyses performed with an anti-LC3 antibody (green). Nuclei were counterstained with DAPI (blue). Scale bar, 50 μm. [file 13046_2020_1692_MOESM3_ESM.tif]

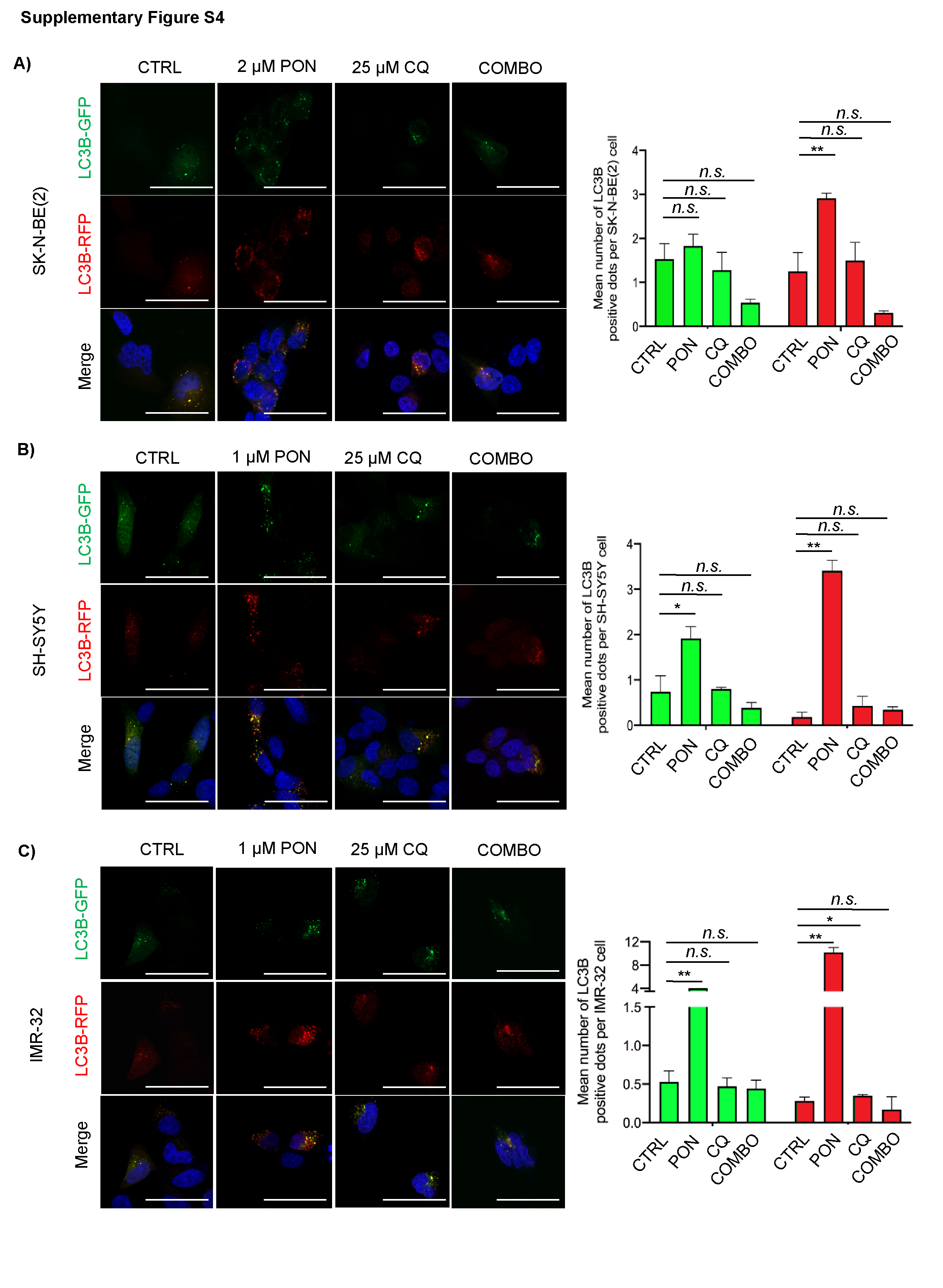

Supplement: Supplementary file 4 — Additional file 4: Supplementary Figure S4. PON stimulates autophagy flux. a) SK-N-BE(2), SH-SY5Y, and IMR-32 neuroblastoma cells expressing an RFP-GFP-LC3 construct were treated for 24 h with the indicated compounds. The neutral pH LC3-positive autophagosomes (GFP and RFP positive dots) and the acidic pH LC3-positive autolysosomes (RFP positive dots) were detected with a confocal microscope. The nuclei were counterstained with Hoechst (blue signal). Scale bar, 50 μm. The quantification of GFP and RFP dots is reported in the histograms on the right. The data are presented as the mean number of positive dots per cell ± SEM. *p < 0.05; **p < 0.01; n.s. – not significant [file 13046_2020_1692_MOESM4_ESM.tif]

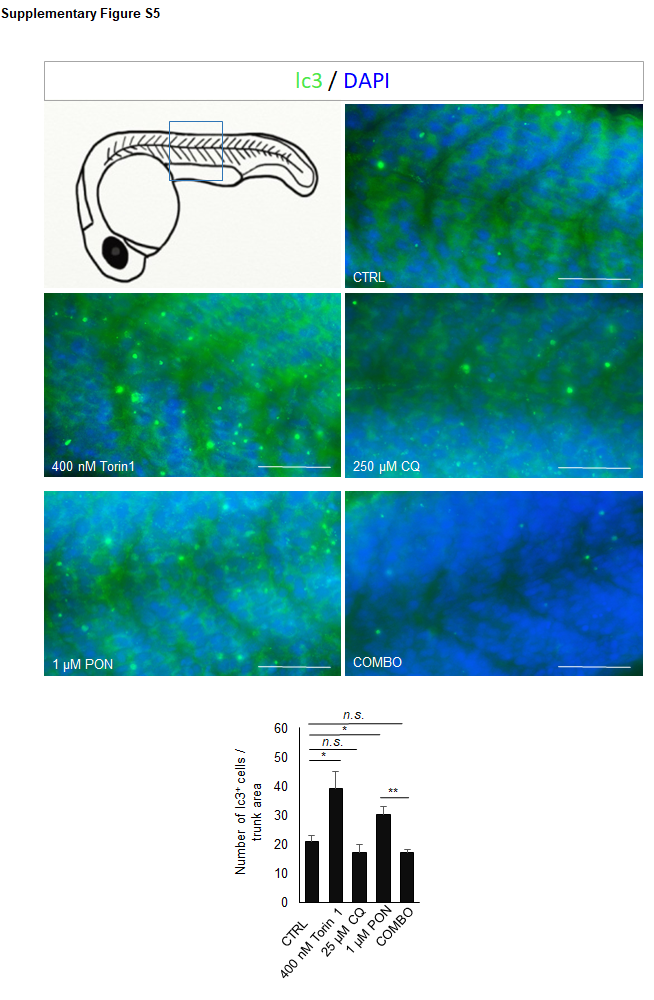

Supplement: Supplementary file 5 — Additional file 5: Supplementary Figure S5. PON treatment activates autophagy in vivo. Representative images show LC3-positive autophagosomes (green puncta) in the trunk area (blue square) of wild-type zebrafish embryos treated as indicated. Nuclei were counterstained with DAPI (blue). A total of 20 embryos was analyzed for each condition per experiment. Scale bars, 50 μm. The data are presented as mean ± SEM of three independent experiments. *p < 0.05; **p < 0.01; n.s. – not significant [file 13046_2020_1692_MOESM5_ESM.tif]

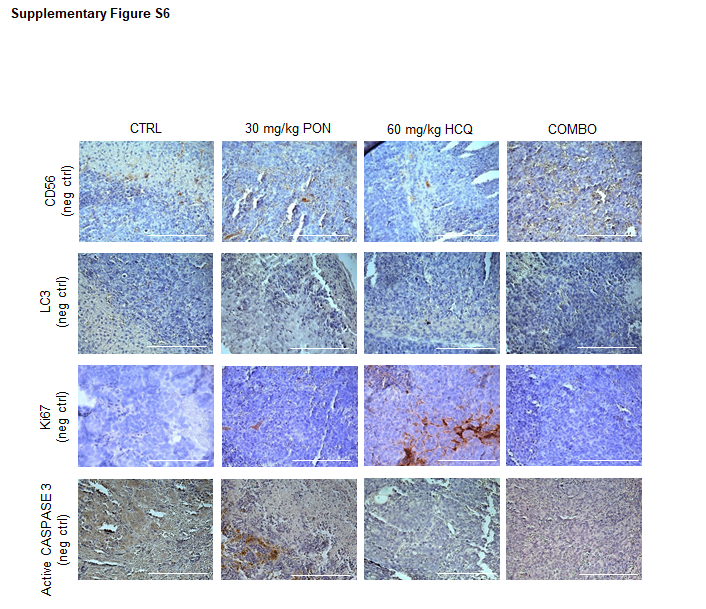

Supplement: Supplementary file 6 — Additional file 6: Supplementary Figure S6. Quality control confirms antibody specificity for immunohistochemical staining. Histopathological examination of tumor sections isolated from nude mice bearing isogenic IMR-32 orthotopic tumors was performed using only the secondary antibodies applied in Fig. 6. Negative controls were primarily used to evaluate the specificity of the immunohistochemistry staining and to identify false-positive staining reactions for each antibody used. Scale bar, 100 μm. [file 13046_2020_1692_MOESM6_ESM.tif]

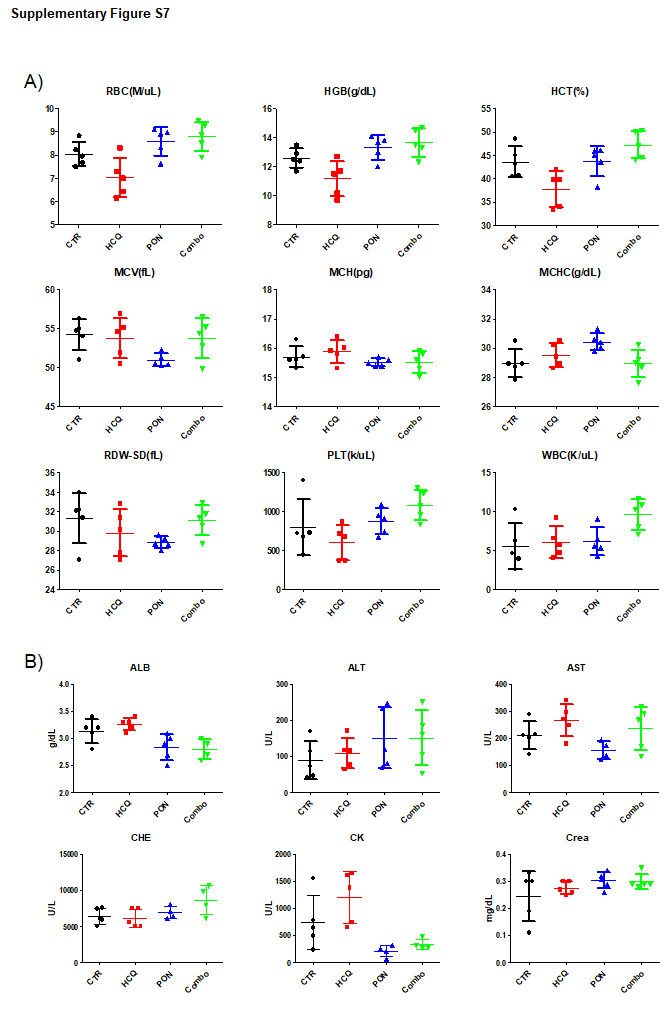

Supplement: Supplementary file 7 — Additional file 7: Supplementary Figure S7. Combination treatment exerts no hematological or clinical chemistry toxicities. IMR-32–bearing mice (n = 5 per group) were treated as reported in Fig. 5 and sacrificed 24 h after the last day of treatment. (A) Red blood cells (RBC), hemoglobin (HGB), hematocrit (HCT), mean cell volume (MCV), mean cell hemoglobin (MCH), mean cell hemoglobin concentration (MCHC), red blood cell distribution width (RDW-SD), platelets (PLT), and white blood cells (WBC) were analyzed. (B) Serum albumin (ALB), glutamic-pyruvic transaminase (ALT), glutamic oxaloacetic transaminase (AST), cholinesterase (CHE), creatine phosphokinase (CK), and creatinine (CREA) were analyzed. [file 13046_2020_1692_MOESM7_ESM.tif]
