# Supplementary material for: Autophagic flux inhibition enhances cytotoxicity of the receptor tyrosine kinase inhibitor ponatinib
Source: J Exp Clin Cancer Res. 2020 Sep 22;39:195. doi: 10.1186/s13046-020-01692-x (PMC7507635; doi:10.1186/s13046-020-01692-x)
Supplement: Supplementary file 8 — Additional file 8: Supplementary Table S1. Genetic background of the neuroblastoma cell lines used in this study. [file 13046_2020_1692_MOESM8_ESM.docx]

**Supplementary Table S1. Genetic background of the neuroblastoma cell lines used in this study.**

| GENE | SH-SY5Y | SK-N-BE(2) | IMR32 | Ref. |
| --- | --- | --- | --- | --- |
| *ATRX* | Wild type | Wild type | Wild type | [1] |
| *MYCN* | Wild type | Amplified | Amplified | [2] |
| *RET* | Wild type | Wild type | Wild type | [3] |
| *ALK* | F1174L | Wild type | Wild type | [4] |

Ref.

1. Zeineldin M, Federico S, Chen X, Fan Y, Xu B, Stewart E, et al. MYCN amplification and ATRX mutations are incompatible in neuroblastoma. Nat Commun. 2020;11:913. Available from: http://www.nature.com/articles/s41467-020-14682-6

2. Thiele CJ. Neuroblastoma Cell Lines. J Hum Cell Cult. 1998;1:21–53.

3. Hofstra RMW, Stulp RP, Stelwagen T, Buys CHCM, Ching Cheng N, Caron H, et al. No mutations found byRET mutation scanning in sporadic and hereditary neuroblastoma. Hum Genet. 1996;97:362–4. Available from: http://link.springer.com/10.1007/BF02185773

4. George RE, Sanda T, Hanna M, Fröhling S, II WL, Zhang J, et al. Activating mutations in ALK provide a therapeutic target in neuroblastoma. Nature. 2008;455:975–8. Available from: http://www.nature.com/articles/nature07397
